# Neuroprotective effects of berberine in preclinical models of ischemic stroke: a systematic review

**DOI:** 10.1186/s40360-025-00843-0

**Published:** 2025-02-21

**Authors:** Ghasem Dolatkhah Laein, Elahe Boumeri, Saghar Ghanbari, Amin Bagherian, Fatemeh Ahmadinasab, Vahid Poudineh, Shima Payandeh, Negar Rashidi

**Affiliations:** 1 Mashhad University of Medical Sciences, Ghasem Abad, Mashhad, Iran; 2 Cognitive Science Media, The Institute for Cognitive Science Studies (ICSS), Tehran, Iran; 3 Clinical Psychology, Islamic Azad University, Semnan, Iran

**Keywords:** Berberine, Ischemic stroke, Systematic review, Preclinical, Animal study, Neuroprotection

## Abstract

**Background/Objective:**

Berberine, a naturally occurring alkaloid, has shown promise as a neuroprotective agent in preclinical models of ischemic stroke. This systematic review aims to comprehensively evaluate the neuroprotective effects of berberine in animal models of cerebral ischemia and elucidate its potential mechanisms of action.

**Methods:**

A systematic search was conducted across nine databases, including PubMed, Embase, Cochrane CENTRAL, Web of Science, Scopus, ScienceDirect, Europe PMC, DOAJ, and Google Scholar, from inception to June 30, 2024. Controlled in vivo studies investigating the neuroprotective effects of berberine in animal models of focal cerebral ischemia were included. Two independent reviewers screened studies, extracted data, and assessed the risk of bias using the SYRCLE tool.

**Results:**

Eighteen studies met the inclusion criteria, encompassing various animal models of ischemic stroke. Berberine treatment consistently resulted in significant reductions in infarct volume and improvements in neurological function compared to control groups. Specifically, berberine doses ranging from 10 mg/kg to 300 mg/kg significantly decreased infarct sizes (*p* < 0.05). Berberine also exhibited anti-inflammatory effects by reducing pro-inflammatory cytokines such as TNF-α, IL-1β, and IL-6, and downregulating the TLR4/NF-κB signaling pathway (*p* < 0.05). Antioxidant effects were evidenced by decreased malondialdehyde levels and increased antioxidant enzymes like superoxide dismutase and glutathione (*p* < 0.05). Additional findings from studies with smaller sample sizes indicated that berberine reduced apoptotic cell death by decreasing TUNEL-positive cells and modulating apoptosis-related proteins, including increasing Bcl-2 and decreasing cleaved caspase-3 levels (*p* < 0.05). Berberine also promoted neurogenesis and synaptic plasticity by increasing the expression of BDNF, TrkB, and synaptic proteins SYP and PSD95 (*p* < 0.05), and enhanced autophagic flux by modulating key autophagy markers (*p* < 0.05). The risk of bias varied among studies, with some lacking detailed reporting on randomization and blinding procedures.

**Conclusion:**

Berberine demonstrates significant neuroprotective effects in preclinical models of ischemic stroke through multiple mechanisms, including anti-inflammatory, antioxidant, anti-apoptotic, and neuroregenerative actions. These findings support the potential of berberine as a multifaceted therapeutic agent for ischemic stroke. Further well-designed clinical trials are warranted to confirm its efficacy and safety in human patients.

**Supplementary Information:**

The online version contains supplementary material available at 10.1186/s40360-025-00843-0.

## Introduction

Stroke remains one of the most significant global health challenges, representing a leading cause of morbidity and mortality worldwide [1]. Ischemic stroke, the most prevalent subtype, accounts for approximately 84.4% of all stroke incidences, as confirmed in a community-based study in Brno, Czech Republic [2]. It occurs due to a sudden disruption of blood flow to a specific area of the brain, initiating a cascade of cellular events that result in neuronal injury and subsequent neurological deficits [3, 4]. Despite considerable advancements in the acute management of ischemic stroke, current therapeutic strategies are limited. The primary treatment involves the administration of tissue plasminogen activator (tPA) within a narrow therapeutic window of 4.5 h post-symptom onset [5]. Given this time constraint, many patients are ineligible for timely intervention, emphasizing the need for alternative neuroprotective therapies that can extend the therapeutic window and provide broader treatment options [6]. Consequently, researchers have increasingly focused on identifying novel neuroprotective agents capable of mitigating the damage caused by ischemic stroke. Berberine, a naturally occurring isoquinoline alkaloid found in various medicinal plants such as Berberis vulgaris (barberry), Coptis chinensis (Chinese goldthread), and Hydrastis canadensis (goldenseal), has emerged as a promising candidate in the treatment of ischemic stroke [7, 8]. It possesses multifaceted pharmacological properties, including potent antioxidant, anti-inflammatory, and anti-apoptotic activities [9–12]. These properties are mediated through several critical molecular pathways, such as the activation of adenosine monophosphate-activated protein kinase (AMPK) and nuclear factor erythroid 2-related factor 2/heme oxygenase-1 (Nrf2/HO-1), which play crucial roles in reducing oxidative stress and inflammation—the primary contributors to neuronal damage during ischemia-reperfusion events [13–15]. Moreover, berberine promotes neuronal survival through the brain-derived neurotrophic factor (BDNF)-TrkB-PI3K/Akt pathway, reducing apoptosis and fostering cell survival, which is essential for neuroprotection in the ischemic brain [16–18]. It also modulates the canopy homolog 2 (CNPY2) signaling pathway, inhibiting apoptosis caused by endoplasmic reticulum stress, thereby preserving neuronal integrity [19]. An additional advantage of berberine is its ability to cross the blood-brain barrier—a challenge for many neuroprotective agents—enhancing its direct action on the brain [20]. Preclinical models have consistently demonstrated that berberine reduces infarct volume and improves neurological outcomes, solidifying its potential as a therapeutic candidate for ischemic stroke [21–23]. Despite this growing body of evidence, there has been no comprehensive systematic review to date that synthesizes these findings and critically evaluates the potential mechanisms of action. Given the limitations of current stroke therapies and the pressing need for effective neuroprotective agents, it is crucial to thoroughly assess berberine’s efficacy and underlying mechanisms in preclinical models. Therefore, the objective of this systematic review is to evaluate the neuroprotective effects of berberine in animal models of ischemic stroke and to elucidate the mechanisms by which it exerts these effects. By critically analyzing the available preclinical studies, we aim to determine whether berberine consistently demonstrates neuroprotective properties that could justify its progression to clinical research.

## Methodology

This systematic review was conducted in accordance with the Preferred Reporting Items for Systematic Reviews and Meta-Analyses (PRISMA) guidelines [24]. Although the review protocol was submitted to the International Prospective Register of Systematic Reviews (PROSPERO) for registration, it was not accepted because the data extraction phase had already commenced at the time of submission. Despite the lack of a registered protocol, we adhered to rigorous methodological standards throughout the process to ensure transparency and reproducibility.

### Search strategy

A comprehensive literature search was performed across Embase, Cochrane Central Register of Controlled Trials (CENTRAL), Web of Science, Scopus, PubMed, ScienceDirect, Europe PMC, and the Directory of Open Access Journals (DOAJ) from inception to June 30, 2024. The search strategy combined controlled vocabulary terms (e.g., MeSH, Emtree) and free-text keywords related to “berberine,” “cerebral ischemia,” and relevant outcome measures. The complete search strategies for each database are provided in Supplementary S1. Reference lists of included studies and relevant reviews were hand-searched to identify additional eligible studies. Conference abstracts from key stroke conferences (International Stroke Conference, European Stroke Organization Conference, and World Stroke Congress) from 2019 to 2024 were screened for potentially relevant studies. Additionally, Google Scholar was searched using the terms “berberine” and “cerebral ischemia” to identify any unpublished or preprint studies.

### Eligibility criteria

We included controlled in vivo experimental studies investigating the neuroprotective effects of berberine in animal models of focal cerebral ischemia. Eligible studies had to meet the following criteria:

- Study Design: Controlled experimental studies involving animals (randomized or non-randomized).
- Animal Models: Studies utilizing focal cerebral ischemia models, such as middle cerebral artery occlusion (MCAO) in rodents.
- Intervention: Administration of berberine as the primary treatment.
- Comparator: A control group receiving no treatment, vehicle (e.g., saline, DMSO), or sham procedure.
- Outcomes: Reporting quantitative data on at least one relevant outcome measure, including infarct volume, neurological deficits, apoptosis, oxidative stress, or inflammation.
- Language: Published in English.

We excluded studies based on the following criteria:

- Study Design: Reviews, meta-analyses, case studies, editorials, letters, conference abstracts, and in vitro studies.
- Animal Models: Studies utilizing global cerebral ischemia models or non-ischemic models.
- Intervention: Studies where berberine was not the primary intervention or was combined with other compounds without separate analysis.
- Language: Non-English publications.

### Study selection and data extraction

The literature search and study selection were independently conducted by two reviewers (GDL and EB) using Covidence systematic review software [25], which streamlined the screening process and ensured consistency. Titles and abstracts were screened to identify potentially eligible studies, followed by full-text assessments for final inclusion. Disagreements were resolved through discussion or consultation with a third reviewer (NR). Data extraction was performed using a standardized form to collect information on study design, animal models, intervention protocols, control procedures, sample sizes, outcome measures, statistical analyses, and quantitative results.

### Risk of bias assessment

Two reviewers (AF and PV) independently assessed the risk of bias for each included study using the SYRCLE risk of bias tool [26], which is specifically designed for animal intervention studies. This tool evaluates selection bias, performance bias, detection bias, attrition bias, reporting bias, and other potential biases. Discrepancies between reviewers were resolved through discussion and consensus, with the involvement of a third reviewer (NR) when necessary.

### Data analysis

Our data analysis plan involved conducting a meta-analysis if sufficient studies assessed the same outcome measures with comparable methodologies. We planned to use random-effects models to account for variability among studies. Heterogeneity would be assessed using the I² statistic, with values greater than 50% indicating substantial heterogeneity. A narrative synthesis will summarize the data qualitatively if meta-analysis is inappropriate due to insufficient studies or heterogeneity.

## Results

### Study selection

**Fig. 1:**
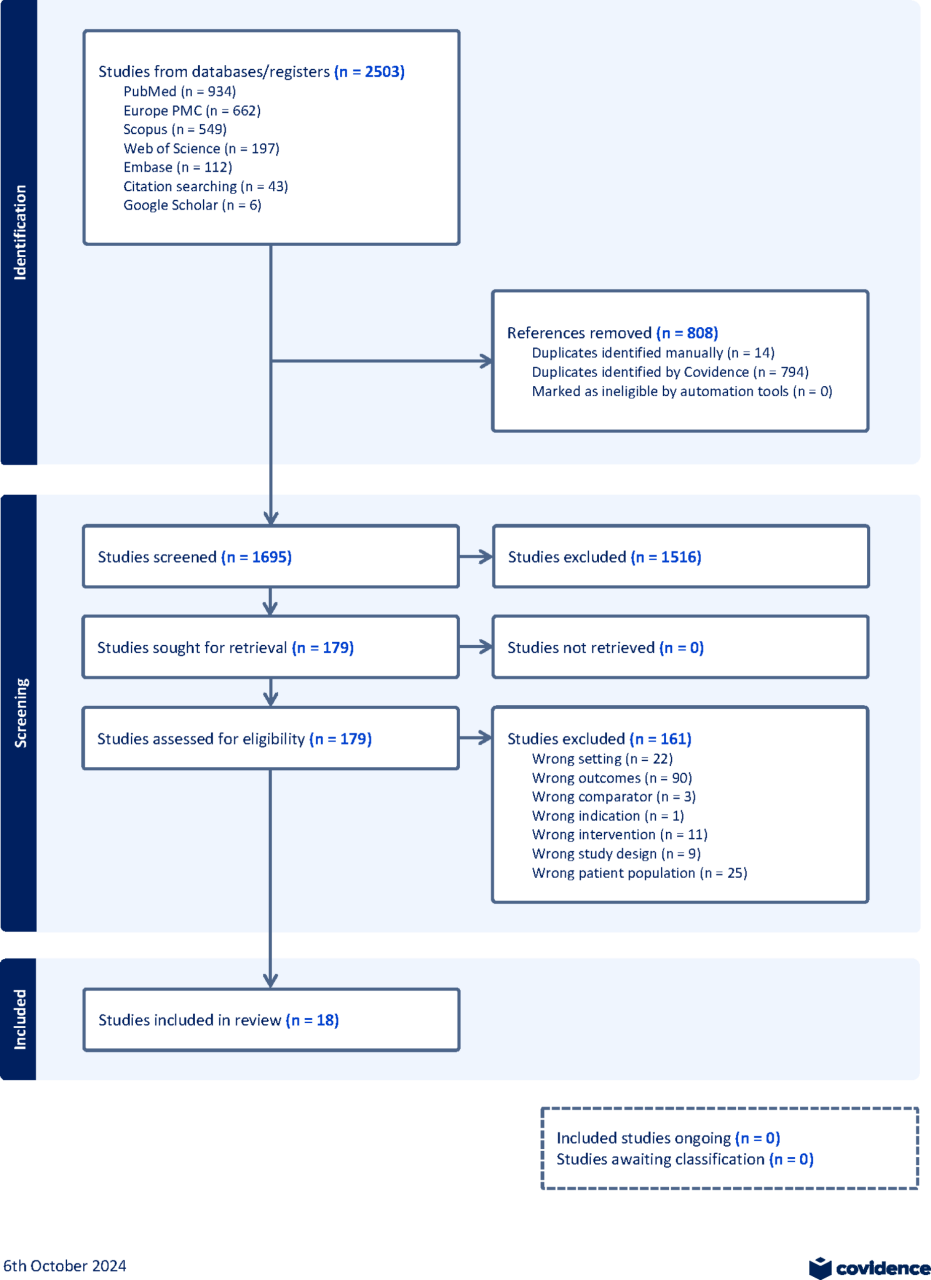
PRISMA flow chart

A total of 2,503 references were imported for screening. After removing 808 duplicates, 1,695 studies were screened against title and abstract, resulting in the exclusion of 1,516 studies. The full-text eligibility assessment of the remaining 179 studies led to the exclusion of 161 studies. Ultimately, 18 studies were included in this systematic review (Fig. 1).

### Study characteristics

A total of 18 studies investigating the effects of berberine on ischemic stroke in animal models were included in this review [27–43]. Table 1 shows Summary of Key Characteristics of Included Studies [27–43]. The studies were conducted between 2008 and 2024, with the majority being performed in China. The study designs were predominantly randomized controlled trials [28–34, 37–39, 42–44], while the remaining studies were controlled trials without explicit randomization [27, 35, 36, 40, 41]. The studies utilized male rodents, predominantly Sprague-Dawley rats and C57BL/6J mice, aged 4 weeks to adult, with body weights ranging from 20 g to 320 g. The focal cerebral ischemia models used were mainly the middle cerebral artery occlusion (MCAO) method, both transient (tMCAO) and permanent (pMCAO), with ischemia durations ranging from 30 min to permanent occlusion and reperfusion times varying from 1 h to 28 days. Berberine administration varied in dosage (10 mg/kg to 300 mg/kg), route (oral gavage, intraperitoneal injections, or intragastric administration), timing (pre-treatment, post-treatment, or both), and duration (single doses to daily administration for up to 28 days). Control groups included vehicle controls (saline, DMSO, or CMC-Na), sham-operated groups, and untreated MCAO groups. Some studies also included additional control groups, such as nimodipine, minocycline, or glycyrrhizin treatment groups [28, 30, 44].

**Table 1:** Summary of key characteristics of included studies

Authors	Study Design	Animal Details						Ischemia Model Details			Intervention Details	Outcomes Assessed
		Species	Strain	Sex	Age	Weight	Number per Group	Type of Focal Ischemia Model	Duration of Ischemia	Reperfusion Time	Berberine Treatment	
Liu et al. [27]	Controlled trial	Rat	Sprague-Dawley	Male	9 weeks	250–280 g	*n* = 6 in several experiments	Middle Cerebral Artery Occlusion/Reperfusion (MCAO/R)	90 min	90 min	Dose: 100 mg/kg/day Route: Oral gavage Timing: Pre-treatment (3 days before MCAO) and once post-MCAO	Infarct Volume, Neurological Deficits, Neuronal Survival/Cell Viability, Autophagy/Lysosomal Pathway Markers.
Yang et al. [28]	RCT	Rat	Sprague-Dawley	N/A	4–5 weeks	180–220 g	mNSS test: *n* = 14 Other assays: *n* = 7	Middle Cerebral Artery Occlusion/Reperfusion (MCAO/R)	120 min	Varied (up to 28 days)	Dose: 100 mg/kg Route: Oral gavage Timing: 30 min before MCAO and daily for 28 days	Infarct Volume, Neurological Deficits, Apoptosis Markers, Neurogenesis/Plasticity Markers, Autophagy/Lysosomal Pathway Markers.
Zhao et al. [29]	RCT	Rat	Sprague-Dawley	Male	8–10 weeks	210–260 g	*n* = 12 per group for the primary grouping (Sham, BBR, CIRI, and CIRI + BBR groups). *n* = 6 per group for additional experiments.	Middle Cerebral Artery Occlusion (MCAO)	120 min	24 h	Dose: 40 mg/kg Route: Oral gavage Timing: 24 h before and 6 h after MCAO	Neurological Deficits, Apoptosis Markers.
Zhu et al. [30]	RCT	Mouse	C57BL/6J	Male	N/A	25–30 g	Initial treatment groups (Sham, Vehicle, Berberine-L, Berberine-H, Glycyrrhizin, Berberine-H + Glycyrrhizin): *n* = 12 per group. Infarct volume and brain edema measurements: *n* = 5 per group. Immunohistochemistry analysis: *n* = 3 per group.	Transient Middle Cerebral Artery Occlusion (tMCAO)	60 min	60 min	Dose: 25 or 50 mg/kg/day Route: Oral gavage Timing: Once daily for 14 days before tMCAO	Infarct Volume, Neurological Deficits, Neuronal Survival/Cell Viability, Brain Edema (Water Content), Inflammatory Cytokines.
Maleki et al. [31]	RCT	Rat	Wistar	Male	10 weeks	200–250 g	*n* = 20 per group for the control group, sham-operated control group, ischemia (MCAO) group, and treatment (MCAO + Berberine) group. *n* = 4 per group for brain water and infarct volume.	Middle Cerebral Artery Occlusion (MCAO)	45 min	24 h reperfusion after ischemia	Dose: 40 mg/kg Route: Not reported Timing: 1 h after ischemia	Infarct Volume, Neurological Deficits, Brain Edema (Water Content), Inflammatory Cytokines, Behavioral/Functional Tests.
Zhu et al. [32]	RCT	Mouse	C57BL/6	Male	N/A	20–24 g	*n* = 6 for groups involved in cerebral blood flow (rCBF) *n* = 12–18 for neurological function tests *n* = 8 for brain infarct volume *n* = 6 for brain water content and histological analysis.	Transient Middle Cerebral Artery Occlusion (tMCAO)	45 min	Varied (up to 28 days)	Dose: 50 mg/kg/day Route: Oral gavage Timing: Before and after surgery (exact timing not specified)	Infarct Volume, Neurological Deficits, Brain Edema (Water Content), Inflammatory Cytokines, Behavioral/Functional Tests.
Duan et al. [33]	RCT	Mouse	C57BL/6J	Male	8–10 weeks	25–30 g	*n* = 18 for Neurological Scores (divided into 3 groups) *n* = 6 for other assays	Middle Cerebral Artery Occlusion/Reperfusion (MCAO/R)	60 min	60 min	Dose: 50 mg/kg Route: Oral gavage Timing: Once daily for 14 days before MCAO/R	Infarct Volume, Neurological Deficits, Neuronal Survival/Cell Viability, Inflammatory Cytokines, Neurogenesis/Plasticity Markers.
Wang et al. [34]	RCT	Mouse	ICR mice	Male	6–8 weeks	N/A	*n* = 5–15 per group	Bilateral Common Carotid Artery Occlusion (BCCAO)	30 min	6–72 h	Dose: Not specified (administered via drinking water) Timing: Daily for 1 week before ischemia	Neuronal Survival/Cell Viability, Behavioral/Functional Tests, Oxidative Stress Markers.
Qichun Zhang et al. [35]	Controlled trial	Rat	Sprague-Dawley	Male	Adult	280–300 g	*n* = 6–8 rats per group	Transient Middle Cerebral Artery Occlusion (MCAO)	120 min	24–72 h	Dose: 40 mg/kg Route: Oral gavage Timing: Single dose 24 h before MCAO	Infarct Volume.
Junjie Zhang et al. [36]	Controlled trial	Rat	Sprague-Dawley	Male	6–8 weeks	300–320 g	*n* = 12 per group	Middle Cerebral Artery Occlusion (MCAO)	N/A	96 h	Dose: 2 mg/kg/day Route: Intraperitoneal injection Timing: After 60 min of reperfusion, daily for 96 h	Infarct Volume, Neurological Deficits, Inflammatory Cytokines.
Liu et al. [37]	RCT	Rat	Sprague-Dawley	Male	N/A	200–240 g	*n* = 10 per group	Middle Cerebral Artery Occlusion/Reperfusion (MCAO/R)	120 min	2–24 h	Dose: 25 mg/kg (BBR-L), 50 mg/kg (BBR-M), 100 mg/kg (BBR-H) Route: Intragastric administration (oral gavage) Timing: Administered once a day for 5 days; first dose 2 h after ischemia/reperfusion	Neurological Deficit, Inflammatory Cytokines, Oxidative Stress Markers, Molecular Pathways.
Q Zhang et al. [38]	RCT	Rat	Sprague-Dawley	Male	Adult	270–300 g	*n* = 3 per group	Middle Cerebral Artery Occlusion (MCAO)	120 min	2–24 h	Dose: 100 mg/kg Route: Intragastrically (oral administration) Timing: Administered 1 h prior to reperfusion (after 2 h of MCAO)	Neuronal Survival/Cell Viability, Apoptosis Markers, Autophagy/Lysosomal Pathway Markers.
Xiaolin Zhang et al. [39]	RCT	Rat	Sprague-Dawley	Male	N/A	250–280 g	*n* = 6 for groups	Permanent Middle Cerebral Artery Occlusion (pMCAO)	Permanent occlusion	N/A	Dose: Low dose (BBR-L): 10 mg/kg High dose (BBR-H): 40 mg/kg Route: Intraperitoneal injection Timing: Administered immediately after cerebral ischemia (post-pMCAO induction)	Infarct Volume, Neurological Deficits, Brain Edema (Water Content).
Zhou et al. [40]	Controlled trial	Mouse	ICR mice	Male	N/A	25–30 g	Neurological: *n* = 11–12 Infarct volume: *n* = 8–9	Middle Cerebral Artery Occlusion (MCAO)	90 min	90 min	Dose: 20 mg/kg Route: Intragastric administration (oral gavage) Timing: First dose: 30 min before reperfusion (90 min after occlusion) Second dose: 1 day after MCAO	Infarct Volume, Neurological Deficits.
Shou et al. [41]	Controlled trial	Mouse	C57BL/6J	Male	6 weeks	N/A	*n* = 6 per group	Middle Cerebral Artery Occlusion (MCAO)	60 min	60 min	Dose: 10, 20, and 50 mg/kg Route: Intragastric (oral gavage) Timing: Treatment started after MCAO surgery; administered once daily for 7 day	Infarct Volume, Neurological Deficits, Behavioral/Functional Tests, Oxidative Stress Markers.
Cao et al. [42]	RCT	Mouse	C57BL/6	Male	Adult	20–25 g	*n* = 3 per group	Middle Cerebral Artery Occlusion (MCAO)	60 min	60 min	Dose: 50 mg/kg per day Route: Intragastric administration (oral gavage) Timing: Administered once daily for 14 consecutive days before MCAO surgery	Inflammatory Cytokines, Molecular Pathways.
Zhang et al. [43]	RCT	Rat	Sprague-Dawley	Male	8 weeks	250–20 g	Berberine group: 40, Control group: 50 (MCAO group)	Middle Cerebral Artery Occlusion (MCAO)	120 min	120 min	Dose: 140 mg/kg Route: Intragastric (i.g.) administration Timing: Pre-treatment (once daily for 7 days before MCAO surgery)	Infarct Volume, Neurological Deficits, Inflammatory Cytokines, Oxidative Stress Markers.
Ni et al. [44]	RCT	Rat	Sprague-Dawley	Male	Adult	280–320 g	*n* = 10 for Sham Control (No tMCAO) *n* = 10 for Model Control (tMCAO) *n* = 10 for tMCAO + Berberine *n* = 10 for tMCAO + Berberine + NBP *n* = 6 for tMCAO + Berberine + Sodium Molybdate *n* = 6 for tMCAO + Berberine + Capsazepine	Transient Middle Cerebral Artery Occlusion (tMCAO)	60 min	60 min	Dose: 30 mg/kg Route: Oral gavage Timing: Administered once daily for 7 days following tMCAO surgery (post-ischemia)	Neurological Deficits, Neuronal Survival/Cell Viability, Inflammatory Cytokines, Oxidative Stress Markers, Neurogenesis/Plasticity Markers, Molecular Pathways.

### Narrative synthesis rationale

Due to the heterogeneity among the included studies and the limited number of studies providing sufficient quantitative data, a formal meta-analysis was not conducted. Specifically, for the “Infarct Volume” outcome, although twelve studies reported on this parameter, only three studies [28, 39, 40] provided adequate quantitative data (means and standard deviations) necessary for meta-analysis. The small number of studies, coupled with variations in animal models, berberine dosages, routes of administration, timing of interventions, and outcome assessment methods, introduced significant heterogeneity. Conducting a meta-analysis under these conditions could lead to unreliable or misleading results due to the potential for substantial statistical heterogeneity and bias [45, 46]. In accordance with the Synthesis Without Meta-analysis (SWiM) guidelines [47], we employed a narrative synthesis approach to systematically summarize and interpret the findings across all outcomes. This approach allows for the integration of evidence from studies with methodological differences and varying outcome measures, providing a comprehensive understanding of berberine’s neuroprotective effects. To provide additional insight into the potential effects of Berberine on infarct volume, effect sizes (Hedges’ g) and 95% confidence intervals were calculated for the three studies with sufficient data [28, 39, 40] using the formula proposed by Hedges and Olkin [48]. These effect sizes and confidence intervals will be presented in the narrative synthesis to complement the qualitative analysis of the findings. Table 2 provides a summary of the direction and significance of Berberine’s effects on the main outcomes across the included studies.

**Table 2:** Direction and significance of Berberine’s effects on outcomes

Authors	Primary Outcomes		Secondary Outcomes					Other outcomes			
	Infarct Volume	Neurological Deficits	Oxidative Stress Markers	Brain Edema (Water Content)	Neuronal Survival/Cell Viability	Behavioral/Functional Tests	Inflammatory Cytokines	Neurogenesis/Plasticity Markers	Apoptosis Markers	Molecular Pathways	Autophagy/Lysosomal Pathway Markers
Liu et al. [27]	↓ *	↓ *	N/A	N/A	↑ **	N/A	N/A	N/A	N/A	N/A	↑ * (Autophagy: LC3-II, Beclin-1), ↓ * (p62, ubiquitin), ↑ * (Cathepsin D, TFEB)
Yang et al. [28]	↓ *** (Cohen’s d = -3.90)	↓ *	N/A	N/A	N/A	N/A	N/A	↑ * (BDNF, TrkB, p-Akt)	↓ *	N/A	↑ * (Autophagy: LC3-II, Beclin-1), ↓ * (p-Akt)
Zhao et al. [29]	N/A	↓ *	N/A	N/A	N/A	N/A	N/A	N/A	↓ *	N/A	N/A
Zhu et al. [30]	↓ *	↓ *	N/A	↓ **	↑ **	N/A	↓ **(Pro-inflammatory)	N/A	N/A	N/A	N/A
Maleki et al. [31]	↓ *	↓ *	N/A	↓ ***	N/A	↑ * (Improved motor function)	↓ **(Pro-inflammatory)	N/A	N/A	N/A	N/A
Zhu et al. [32]	↓ *	↓ *	N/A	↓ *	N/A	↑ ** (Improved motor function)	↓ *(Pro-inflammatory)	N/A	N/A	N/A	N/A
Duan et al. [33]	↓ *	↓ *	N/A	N/A	↑ *	N/A	↓ *(Pro-inflammatory)	↑ * (SYP, PSD95)	N/A	N/A	N/A
Wang et al. [34]	N/A	N/A	↓ *** (MDA), ↑ ** (GSH, SOD)	N/A	↑ **	↓ * (Reduced anxiety)	N/A	N/A	N/A	N/A	N/A
Qichun Zhang et al. [35]	↓ *	N/A	N/A	N/A	N/A	N/A	N/A	N/A	N/A	N/A	N/A
Junjie Zhang et al. [36]	↓ *	↓ *	N/A	N/A	N/A	N/A	↓ *(Pro-inflammatory)	N/A	N/A	N/A	N/A
Liu et al. [37]	N/A	N/A	↓ * (MDA), ↑ * (SOD)	N/A	↑ *	N/A	↓ *(Pro-inflammatory)	N/A	N/A	↓ * (TLR4, NF-κB)	N/A
Q Zhang et al. [38]	N/A	N/A	N/A	N/A	↑ *	N/A	N/A	N/A	↓ *	N/A	↓ ** (HIF-1α, p53)
Xiaolin Zhang et al. [39]	↓ * (Cohen’s d = -1.69)	↓ *	N/A	↓ *	N/A	N/A	N/A	N/A	N/A	N/A	N/A
Zhou et al. [40]	↓ * (Cohen’s d = -1.04)	↓ **	N/A	N/A	N/A	N/A	N/A	N/A	N/A	N/A	N/A
Shou et al. [41]	↓ *	↓ *	↓ ** (ROS), ↑ * (GSH)	N/A	N/A	↑ ** (Improved motor function)	N/A	N/A	N/A	N/A	N/A
Cao et al. [42]	N/A	N/A	N/A	N/A	N/A	N/A	↓ * (Pro-inflammatory), ↑ * (Anti-inflammatory)	N/A	N/A	↓ * (Malat1, HMGB1)	N/A
Zhang et al. [43]	↓ *	↓ *	↓ * (MDA), ↑ * (Antioxidants: GSH, GPx, CAT, SOD)	N/A	N/A	N/A	↓ **(Pro-inflammatory)	N/A	N/A	N/A	N/A
Ni et al. [44]	N/A	↓ *	↓ ** (MDA), ↑ ** (H₂S, GSH)	N/A	↑ **	N/A	↓ ** (Pro-inflammatory), ↑ ** (Anti-inflammatory)	↑ * (c-fos, ChAT)	N/A	↑ * (Vagal nerve activity), ↓ * (HMGB1, Malat1)	N/A

Ps: ↑: Increase, ↓: Decrease, *: *p* < 0.05, **: *p* < 0.01, ***: *p* < 0.001, N/A: Not Assessed

### Primary outcomes

#### Effects of berberine on infarct volume

**Fig. 2:**
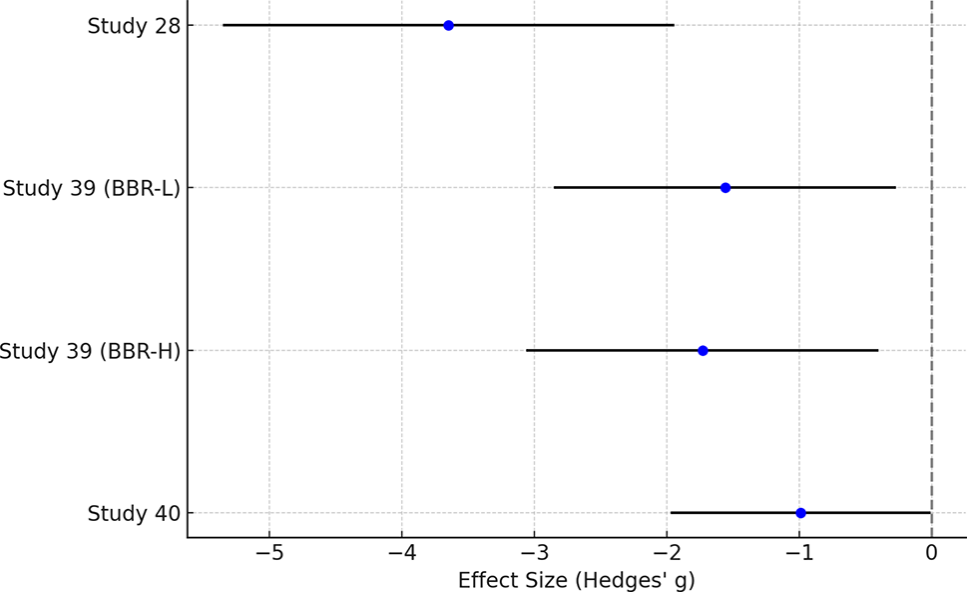
Forest plot of effect sizes (Hedges’ g) and 95% confidence intervals for Berberine’s impact on infarct volume in animal models of ischemic stroke

Twelve studies consistently demonstrated that berberine treatment significantly reduced infarct volume compared to control groups. The reduction in infarct volume was statistically significant, with p-values ranging from *p* < 0.05 to *p* < 0.001 [27, 28, 30–33, 35, 36, 39–41, 43]. The effect sizes, calculated using Cohen’s d with pooled standard deviations, were reported for the three studies with sufficient data [28, 39, 40]. Figure 2 presents the effect sizes (Hedges’ g) and 95% confidence intervals for these three studies [28, 39, 40]. In one study, berberine-treated animals exhibited a substantial reduction in infarct volume. The mean infarct volume in the berberine group was 15.6% (SD = 3.2%, *n* = 7), compared to 36.3% (SD = 6.8%, *n* = 7) in the control group. The calculated Hedges’ g was − 3.65 with a 95% confidence interval (CI) of -5.35 to -1.94, indicating a large effect size favoring berberine treatment [28]. In second study, two doses of berberine were tested: a low dose (BBR-L) and a high dose (BBR-H). The BBR-L group had a mean infarct volume of 34.73% (SD = 2.63%, *n* = 6), and the BBR-H group had 30.93% (SD = 6.66%, *n* = 6), compared to the control group’s 44.17% (SD = 7.44%, *n* = 6). The Hedges’ g for the BBR-L group was − 1.56 (95% CI: -2.85 to -0.27), and for the BBR-H group, it was − 1.73 (95% CI: -3.06 to -0.40), both representing large effect sizes [39]. A third study, berberine treatment resulted in a mean infarct volume of 14.98 mm³ (SD = 21.37 mm³, *n* = 9), significantly lower than the control group’s 65.9 mm³ (SD = 66.15 mm³, *n* = 9). The Hedges’ g was − 0.99 with a 95% CI of -1.97 to -0.01, indicating a large effect size favoring berberine [40]. These findings across multiple studies underscore the significant impact of berberine in reducing infarct volume, demonstrating a consistent treatment effect across different dosing regimens and measurement methods.

#### Effects of berberine on neurological deficits

Berberine treatment significantly reduced neurological deficit scores in animal models of ischemic stroke, as assessed by the Modified Neurological Severity Score (mNSS) [27–29, 44] and variations of the Longa scoring system [30–33, 39–41, 43]. The mNSS ranges from 0 (no deficit) to 18 (most severe deficits) [27–29, 44], while the Longa scale typically ranges from 0 (no deficit) to 4 (severe deficits) [30, 32, 33, 39, 43]. Other assessment methods included a six-point behavioral scale [31], a five-point neurological deficit scoring scale [36], and a four-tiered grading system [40]. Berberine’s neuroprotective effects were observed as early as 24 h post-ischemia [27, 29–31, 33, 36, 39, 43] and persisted up to 28 days in some studies [28, 32, 41, 44]. Sample sizes per group ranged from 6 [27, 31, 39, 43] to 18 [33], with 12 being the most common [29, 30, 32, 40]. Berberine treatment significantly reduced neurological deficit scores compared to control/model groups in all studies reporting statistical significance (*p* < 0.05 to *p* < 0.001) [28, 29, 31–33, 36, 39–41, 43]. Higher doses of berberine (e.g., 50 mg/kg) tended to show greater neuroprotective effects compared to lower doses (e.g., 25 mg/kg) [30, 39].

### Secondary outcomes

#### Effects on inflammatory cytokines

Berberine significantly decreased serum levels of pro-inflammatory cytokines, including tumor necrosis factor-alpha (TNF-α) interleukin-1 beta (IL-1β), and interleukin-6 (IL-6), compared to vehicle or control groups (*P* < 0.01 or *P* < 0.05) [30–33, 36, 37, 42–44]. Additionally, berberine increased the levels of anti-inflammatory cytokines, such as interleukin-10 (IL-10), transforming growth factor-beta 1 (TGF-β1), and interleukin-4 (IL-4) [31, 32, 42, 44]. Berberine also downregulated the mRNA expression levels of inflammatory mediators, including high mobility group box 1 (HMGB1), toll-like receptor 4 (TLR4), nuclear factor-kappa B (NF-κB), TNF-α, IL-1β, and IL-6, compared to vehicle groups (*P* < 0.01) [30, 43]. Furthermore, berberine decreased the protein expression levels of HMGB1, TLR4, and NF-κB, as demonstrated by immunohistochemistry and western blotting (*P* < 0.01) [30, 31]. The sample sizes in these studies ranged from 3 to 10 animals per group [30–32, 36, 37, 42–44]. The effects of berberine on inflammatory cytokines were dose-dependent, with higher doses showing more significant reductions in pro-inflammatory cytokines and increases in anti-inflammatory cytokines [37, 44].

#### Effects on neuronal survival and cell viability

Berberine’s neuroprotective effects on neuronal survival and cell viability were assessed using various methods, including Nissl staining [27, 34], Fluoro-Jade C (FJC) staining [27], immunofluorescence [27, 44], hematoxylin and eosin (H&E) staining [30, 34, 38], NeuN immunohistochemistry [44], Western blot analysis [33], and Modified Neurological Severity Score (mNSS) [37]. Berberine significantly increased neuron survival compared to control groups [27, 30, 34, 38, 44], with fewer injured neurons observed in berberine-treated groups (*p* < 0.01) [30]. Higher doses of berberine demonstrated greater neuroprotective effects compared to lower doses (*p* < 0.05) [30]. Berberine also significantly reduced NeuN-positive neuronal loss compared to tMCAO group (*p* < 0.05, *p* < 0.01) [44]. Additionally, berberine treatment reduced the expression of Iba-1, GFAP, and NLRP3 inflammasome compared to control [33], and exhibited reduced nuclear pyknosis and increased Nissl bodies compared to CIRI group [34]. In one study, the berberine-treated group (BBR-H, 100 mg/kg) had significantly lower mNSS scores than the Model Group (*p* < 0.05) [37]. Berberine’s neuroprotective effects were statistically significant compared to control groups, with p-values ranging from *p* < 0.05 to *p* < 0.001 [27, 30, 37, 44].

#### Effects on oxidative stress markers

Five studies reported the effects of berberine on oxidative stress markers in animal models of ischemic stroke [34, 37, 41, 43, 44]. Berberine treatment significantly decreased malondialdehyde (MDA) levels (*P* < 0.05) [34, 37, 43] and increased glutathione (GSH) levels (*P* < 0.05) [34, 43] compared to control groups. Reactive oxygen species (ROS) levels were significantly reduced in berberine-treated animals, as evidenced by decreased intensity of red fluorescence in dihydroethidium (DHE) staining (*P* < 0.0001) [34] and reduced H2DCFDA fluorescence (*P* < 0.05, *P* < 0.01) [41]. One study found that berberine dose-dependently improved superoxide dismutase (SOD) activity and decreased MDA levels compared to the model group (*P* < 0.05) [37]. Additionally, berberine significantly increased H₂S content in feces (*P* < 0.05), with H₂S production linked to sulfate-reducing bacteria [44]. Berberine also significantly improved other oxidative stress markers, including nitric oxide (NO), glutathione disulfide (GSSG), Mn-SOD, Cu/Zn-SOD, catalase (CAT), and glutathione peroxidase (GPx), compared to control groups (*P* < 0.05) [43].

#### Effects on behavioral and functional outcomes

Four studies evaluated the effects of berberine on behavioral and functional outcomes in animal models of ischemic stroke [31, 32, 34, 41]. The ladder test, performed 24 h after reperfusion, showed that berberine significantly improved motor function compared to the ischemia group (*P* < 0.05) [31]. In the corner test, berberine treatment resulted in a lower number of right turns on days 1 (*P* < 0.01), 7 (*P* < 0.05), and 14 (*P* < 0.05) post-stroke compared to vehicle controls [32, 41]. The rotarod test demonstrated that berberine significantly increased latency to fall on days 7 (*P* < 0.05), 14 (*P* < 0.01), and 28 (*P* < 0.01) after stroke [32], and on day 7 (*P* < 0.05, *P* < 0.01) in another study [41]. Anxiety-like behaviors were assessed 72 h post-reperfusion using the elevated plus-maze test, open field test, and light/dark box test [34]. Berberine significantly increased the frequency and time spent in the open arms (*P* < 0.05) and central area (*P* < 0.05) in the elevated plus-maze and open field tests, respectively. In the light/dark box test, berberine decreased time spent in the dark box and increased transitions between compartments (*P* < 0.05, *P* < 0.01) [34].

#### Effects on brain edema (water content)

Berberine treatment significantly reduced brain edema, as measured by the wet-dry weight method, in animal models of ischemic stroke [30–32, 39]. At 24 h after reperfusion or tMCAO, berberine-treated groups exhibited lower brain water content compared to control groups (*p* < 0.05) [30–32, 39]. In one study, the mean brain water content was 82.61% ± 0.40% in the vehicle group, while it was 81.28% ± 0.35% (*p* < 0.01) and 80.23% ± 0.46% (*p* < 0.01) in the low-dose and high-dose berberine groups, respectively [30]. The high-dose berberine group also showed a significantly lower brain water content compared to the low-dose group (*p* < 0.05) [30]. Another study reported brain water content values of 77.35% in the control group, 78.83% in the sham group, 80.63% in the ischemia group, and 77.89% in the berberine-treated group (*p* < 0.01 ischemia vs. control; *p* < 0.001 berberine vs. ischemia) [31]. The sample sizes in these studies ranged from 5 to 6 animals per group [30, 39], providing a reliable basis for the conclusions. The consistent findings across multiple studies demonstrate that berberine effectively mitigates brain edema in ischemic stroke models, likely contributing to its overall neuroprotective effects. These results suggest that berberine may have potential as a therapeutic agent for reducing brain edema and improving outcomes in ischemic stroke patients.

### Risk of bias assessment

The risk of bias in the included studies was assessed using SYRCLE’s risk of bias tool [26]. Supplementary S2 presents the detailed risk of bias assessment for each study across the domains. Of the 18 studies, 10 (55.6%) were judged to have a low risk of bias [28–32, 34, 38, 39, 43, 44], while 3 (16.7%) had a high risk of bias [27, 36, 40], and 5 (27.8%) had an unclear risk of bias [33, 35, 37, 41, 42]. The main sources of bias were inadequate reporting of allocation concealment, blinding of personnel and outcome assessors, and handling of incomplete outcome data. The potential impact of these biases on the study results will be considered in the interpretation of the findings.

## Discussion

This systematic review provides a comprehensive synthesis of preclinical studies investigating the neuroprotective effects of berberine in animal models of ischemic stroke. By analyzing 18 studies conducted between 2008 and 2024, we have compiled evidence that underscores berberine’s potential in mitigating ischemic brain injury. The findings consistently demonstrate that berberine treatment leads to significant reductions in infarct volume and improvements in neurological function. Moreover, berberine exhibits beneficial effects on inflammatory processes, oxidative stress, neuronal survival, behavioral outcomes, and brain edema. Although outcomes related to apoptosis markers, molecular pathways, neurogenesis and plasticity markers, and autophagy/lysosomal pathway markers were assessed in only three studies each, their findings offer valuable insights into berberine’s neuroprotective mechanisms. Berberine significantly reduced TUNEL-positive cells compared to control groups (*p* < 0.05) [28, 29, 38], indicating decreased apoptotic cell death. It increased anti-apoptotic Bcl-2 levels and decreased the cleaved caspase-3/caspase-3 ratio (*p* < 0.05) [28], while no significant changes were observed in pro-apoptotic Bax levels [28]. In terms of molecular pathways, berberine reduced TLR4 and NF-κB/p65 protein and mRNA levels elevated by ischemia (*p* < 0.05) [37], modulated the Malat1/miR-181c-5p/HMGB1 axis by reducing Malat1 and HMGB1 expression [42], and increased vagal nerve activity post-MCAO (*p* < 0.05, *p* < 0.01) [44]. Regarding neurogenesis, berberine increased BDNF, TrkB, and p-Akt expression compared to the MCAO group (*p* < 0.05) [28], and elevated SYP and PSD95 levels [33], suggesting enhanced neuroplasticity. In autophagy pathways, berberine enhanced autophagic flux by increasing the LC3-II/I ratio and Beclin-1 expression and decreasing p62 and ubiquitin levels [27]; these effects were reversed by autophagy inhibition with bafilomycin A1 [27]. Although limited by the small number of studies, these findings indicate that berberine exerts neuroprotective effects through anti-apoptotic actions, modulation of inflammatory and genetic pathways, promotion of neurogenesis, and regulation of autophagy. Comparing our results with those of Luo et al. [49], who conducted a meta-analysis on the effects of adjuvant berberine therapy in acute ischemic stroke patients, we observe a convergence of evidence supporting berberine’s efficacy. Luo et al. demonstrated that berberine, when combined with conventional treatments, significantly reduced inflammatory markers such as hs-CRP, IL-6, and TNF-α, and improved neurological outcomes measured by the NIHSS score. These clinical findings align with our preclinical results, where berberine administration led to significant decreases in pro-inflammatory cytokines and improvements in neurological deficit scores. Similarly, the review by Sunhe et al. [50] provides an extensive overview of berberine’s neuroprotective mechanisms across various central nervous system diseases, including ischemic stroke. They highlight berberine’s ability to modulate oxidative stress, neuroinflammation, apoptosis, and autophagy pathways. Our systematic review corroborates these mechanisms, as we found that berberine reduced oxidative stress markers like MDA and ROS, and modulated apoptosis-related proteins, contributing to neuronal survival. Additionally, our findings are supported by Zhao et al. [19], who demonstrated that berberine attenuates neuronal apoptosis by inhibiting the CNPY2-mediated endoplasmic reticulum stress (ERS) pathway in a cerebral ischemia-reperfusion injury model. They reported that berberine reduced TUNEL-positive cells and decreased the expression of apoptosis-related proteins such as caspase-3, GRP78, CHOP, and p-PERK/PERK. This aligns with our observation that berberine exerts anti-apoptotic effects by modulating ERS pathways, further elucidating its neuroprotective mechanisms in ischemic stroke. Hao et al. [51] conducted a systematic review and meta-analysis focusing on berberine’s neuroprotective effects in diabetes-related cognitive impairment. Their findings underscore berberine’s efficacy in improving cognitive function, reducing oxidative stress, and attenuating inflammation in diabetic animal models. While their study centers on a different pathological condition, the underlying mechanisms - such as antioxidant and anti-inflammatory effects - are consistent with those observed in our review. This suggests that berberine’s neuroprotective properties may have broad applicability across various neurological disorders involving oxidative stress and inflammation. Our systematic review extends the current understanding of berberine by specifically focusing on its effects in ischemic stroke models. The consistent reduction in infarct volume across multiple studies is particularly noteworthy. For instance, berberine doses ranging from 10 mg/kg to 300 mg/kg significantly decreased infarct sizes [27, 28, 30–33, 35, 36, 39–41, 43], indicating a dose-responsive relationship. This effect is critical because infarct volume is a primary determinant of neurological outcome post-stroke. In terms of neurological function, berberine-treated animals showed significant improvements in behavioral tests, such as the Modified Neurological Severity Score and motor function assessments [27–33, 39–41, 43, 44]. These functional outcomes are essential for translating preclinical findings into clinical relevance, as they reflect potential improvements in patient mobility and independence post-stroke. The anti-inflammatory effects of berberine observed in our review are consistent with those reported by Luo et al. [49] and Sunhe et al. [50]. Berberine’s ability to decrease pro-inflammatory cytokines and inhibit key inflammatory pathways like NF-κB suggests a robust mechanism by which it can mitigate secondary injury processes following ischemic stroke. Specifically, berberine significantly decreased levels of TNF-α, IL-1β, and IL-6 [30–33, 36, 37, 42–44], and downregulated the expression of TLR4 and NF-κB [30, 31, 43]. Oxidative stress is another critical factor in ischemic injury, and our review found that berberine effectively reduces oxidative damage by decreasing MDA levels [34, 37, 41, 43, 44], reducing ROS production [34], and enhancing antioxidant defenses like SOD and GSH [34, 37, 43]. This antioxidative action complements its anti-inflammatory effects, providing a comprehensive neuroprotective profile that addresses multiple facets of ischemic injury. While our review demonstrates promising results, it is important to acknowledge the limitations inherent in preclinical studies. Variations in animal models, berberine dosages, administration routes, and timing introduce heterogeneity that may affect the generalizability of the findings. Additionally, some studies lacked detailed reporting on methodological aspects such as randomization and blinding, which could introduce bias [27, 35, 36, 40, 41]. These omissions increase the risk of bias and may overestimate the reported effects of berberine. Small sample sizes in some studies may reduce the statistical power and reliability of the results, potentially affecting the validity of the conclusions drawn.

Furthermore, the limited number of studies assessing specific mechanisms, such as apoptosis markers, autophagy pathways, and neurogenesis markers, restricts our understanding of the comprehensive mechanisms by which berberine exerts its neuroprotective effects. Most studies focused on short-term outcomes, with few evaluating long-term functional recovery and neurobehavioral performance, which are crucial for translating preclinical findings into clinical applications. Moreover, publication bias may be a concern, as studies with negative or null results are less likely to be published, potentially skewing the overall assessment of berberine’s efficacy. The predominance of positive findings in the literature may overrepresent the true effect size.

To address these limitations, future research should aim for greater standardization in experimental designs. Employing consistent animal models with well-defined parameters, standardized dosing regimens, and uniform outcome measures will enhance comparability and reproducibility of results. Detailed reporting of methodological procedures, including randomization, blinding, and sample size calculations, is essential to reduce bias and improve the quality of evidence. Investigating the optimal dosing strategies, including dose-response relationships and the timing of berberine administration relative to ischemic events, will help in optimizing treatment protocols. Exploring different routes of administration and formulations may improve bioavailability and therapeutic efficacy. Expanding research into the specific molecular mechanisms underlying berberine’s neuroprotective effects is also necessary. Studies focusing on detailed analyses of apoptosis, autophagy, neurogenesis, and other relevant pathways will deepen our understanding and may identify novel therapeutic targets. Assessing long-term outcomes and functional recovery through extended follow-up periods and comprehensive neurobehavioral testing will provide insights into the sustained benefits of berberine treatment. Moreover, addressing potential publication bias by encouraging the publication of negative findings and registering preclinical studies in databases can contribute to a more balanced evidence base. Collaborative efforts and multi-center studies may also enhance the robustness of future research.

## Conclusion

In conclusion, our systematic review provides comprehensive preclinical evidence that berberine exerts significant neuroprotective effects in ischemic stroke models. The consistency of these effects across studies and their alignment with clinical findings highlight berberine’s potential as a multifaceted therapeutic agent. These findings warrant further investigation to fully elucidate berberine’s role in the treatment of ischemic stroke and potentially other neurodegenerative conditions.

## Electronic supplementary material

Below is the link to the electronic supplementary material.

Supplementary Material 1

Supplementary Material 2

## Data Availability

Data is provided within the manuscript or supplementary information files.

## Abbreviations

tPA: Tissue Plasminogen Activator
AMPK: Adenosine Monophosphate-Activated Protein Kinase
Nrf2/HO-1: Nuclear Factor Erythroid 2-Related Factor 2/Heme Oxygenase-1
BDNF: Brain-Derived Neurotrophic Factor
TrkB: Tropomyosin Receptor Kinase B
PI3K/Akt: Phosphoinositide 3-Kinase/Protein Kinase B (Akt)
CNPY2: Canopy Homolog 2
BBB: Blood-Brain Barrier
MCAO: Middle Cerebral Artery Occlusion
tMCAO: Transient Middle Cerebral Artery Occlusion
pMCAO: Permanent Middle Cerebral Artery Occlusion
DMSO: Dimethyl Sulfoxide
CMC-Na: Carboxymethylcellulose Sodium
SWiM: Synthesis Without Meta-analysis
CI: Confidence Interval
mNSS: Modified Neurological Severity Score
TNF-α: Tumor Necrosis Factor-alpha
IL-1β: Interleukin-1 Beta
IL: Interleukin
TGF-β1: Transforming Growth Factor-beta 1
mRNA: Messenger Ribonucleic Acid
HMGB1: High Mobility Group Box 1
TLR4: Toll-Like Receptor 4
NF-κB: Nuclear Factor-kappa B
FJC: Fluoro-Jade C
H&E: Hematoxylin and Eosin
NeuN: Neuronal Nuclei
Iba-1: Ionized Calcium-Binding Adaptor Molecule 1
GFAP: Glial Fibrillary Acidic Protein
NLRP3: NOD-Like Receptor Family Pyrin Domain Containing 3
CIRI: Cerebral Ischemia-Reperfusion Injury
MDA: Malondialdehyde
GSH: Glutathione
ROS: Reactive Oxygen Species
DHE: Dihydroethidium
H₂DCFDA: 2’,7’-Dichlorodihydrofluorescein Diacetate
SOD: Superoxide Dismutase
H₂S: Hydrogen Sulfide
NO: Nitric Oxide
GSSG: Glutathione Disulfide
Mn-SO: Manganese Superoxide Dismutase
Cu/Zn-SOD: Copper/Zinc Superoxide Dismutase
CAT: Catalase
GPx: Glutathione Peroxidase
TUNEL: Terminal deoxynucleotidyl transferase dUTP nick-end labeling
Bcl-2: B-cell lymphoma 2
Bax: Bcl-2-associated X protein
NF-κB/p65: Nuclear Factor kappa-light-chain-enhancer of activated B cells / p65 subunit
Malat1: Metastasis-Associated Lung Adenocarcinoma Transcript 1
miR-181c-5p: MicroRNA-181c-5p
p-Akt: Phosphorylated Protein Kinase B (Akt)
SYP: Synaptophysin
LC3-II/I: Microtubule-Associated Protein 1 Light Chain 3-II/I Ratio
Beclin-1: Beclin-1 (Autophagy-Related Protein)
p62: Sequestosome 1 (also known as p62)
hs-CRP: High-Sensitivity C-Reactive Protein
NIHSS: National Institutes of Health Stroke Scale
