# Supplementary material for: Neuroprotective effects of berberine in preclinical models of ischemic stroke: a systematic review
Source: BMC Pharmacol Toxicol. 2025 Feb 21;26:40. doi: 10.1186/s40360-025-00843-0 (PMC11844076; doi:10.1186/s40360-025-00843-0)
Supplement: Supplementary file 2 — Supplementary Material 2 [file 40360_2025_843_MOESM2_ESM.docx]

**Title: Neuroprotective effects of berberine in preclinical models of ischemic stroke: A systematic review**

**Comprehensive Search Approach**

In 2020, our research team initiated a study to investigate the neuroprotective effects of berberine in animal models of ischemic stroke. However, due to the COVID-19 pandemic, we were forced to halt our research. In October 2023, we reconvened to resume our work and continue the study using the COVIDENCE platform for systematic review management.

Throughout the course of our research, we encountered challenges that required us to modify our search criteria three times to ensure a comprehensive and up-to-date literature search. Our primary objective was to identify all relevant studies that explored the use of berberine for neuroprotection in animal models of ischemic stroke.

To achieve this goal, we conducted an extensive literature search across nine major biomedical databases:

1. PubMed

2. ScienceDirect

3. Scopus

4. Web of Science

5. Europe PMC

6. Directory of Open Access Journals (DOAJ)

7. Google Scholar

8. Embase

9. Cochrane Central Register of Controlled Trials

The decision to search across multiple databases was driven by the understanding that each database has its own unique coverage and indexing practices. By leveraging the complementary nature of these databases, we aimed to combat database bias and minimize the risk of overlooking pertinent studies. This approach ensures a more comprehensive identification of relevant preclinical research on berberine for neuroprotection in animal models of ischemic stroke. Although our search strategy may not be exhaustive, given the constantly evolving nature of biomedical research, we believe that our expansive cross-database approach provides a strong foundation for identifying the most relevant and up-to-date studies in this field. As we progress with our systematic review, we will carefully screen and assess the identified studies using predefined inclusion and exclusion criteria. This process will help us to select the most appropriate and high-quality studies that can contribute to our understanding of the neuroprotective effects of berberine in animal models of ischemic stroke.

PubMed

("berberine"[MeSH Terms] OR "berberine"[All Fields] OR "isoquinolines"[MeSH Terms] OR "isoquinolines"[All Fields] OR "isoquinoline"[All Fields] OR "berberis"[MeSH Terms] OR "berberis"[All Fields] OR "coptis"[MeSH Terms] OR "coptis"[All Fields] OR "Berberine"[tiab] OR "Hydrastis"[tiab] OR "Mahonia"[tiab] OR "Phellodendron"[tiab] OR "Barberry"[tiab] OR "Goldenseal"[tiab] OR "Goldthread"[tiab] OR "Oregon Grape"[tiab] OR "Amur Cork Tree"[tiab] OR "Huang Lian"[tiab] OR "Huang Bai"[tiab] OR "Huang Qin"[tiab] OR Berberine[nm] OR Berberine[tw] OR "2086-83-1"[rn] OR "Isoquinoline Alkaloid"[tiab] OR "Protoberberine"[tiab] OR Dimethoxyberbine[tiab] OR Berbericinine[tiab] OR "Berberrubine"[tiab] OR "Palmatine"[tiab] OR "Jatrorrhizine"[tiab] OR "Coptisine"[tiab] OR "Columbamine"[tiab] OR "Berberastine"[tiab] OR "Oxyberberine"[tiab] OR "Canadine"[tiab]) AND ("brain ischemia"[MeSH Terms] OR ("brain"[All Fields] AND "ischemia"[All Fields]) OR "brain ischemia"[All Fields] OR ("cerebral"[All Fields] AND "ischemia"[All Fields]) OR "cerebral ischemia"[All Fields] OR "brain infarction"[MeSH Terms] OR ("brain"[All Fields] AND "infarction"[All Fields]) OR "brain infarction"[All Fields] OR ("cerebral"[All Fields] AND "infarction"[All Fields]) OR "cerebral infarction"[All Fields] OR (middle[All Fields] AND cerebral[All Fields] AND artery[All Fields] AND occlusion[All Fields]) OR mcao[All Fields] OR (brain[All Fields] AND ischemia[All Fields] AND transient[All Fields]) OR (transient[All Fields] AND cerebral[All Fields] AND ischemia[All Fields]) OR "transient cerebral ischemia"[All Fields] OR "Cerebrovascular Disorders"[MeSH] OR "Stroke"[MeSH] OR "Ischemic Stroke"[tiab] OR "Cerebrovascular Accident"[tiab] OR "Transient Ischemic Attack"[tiab] OR "Focal Cerebral Ischemia"[tiab] OR "Cerebral Hypoperfusion"[tiab] OR "Cerebrovascular Insufficiency"[tiab] OR "Cerebrovascular Ischemia"[tiab] OR Stroke[tiab] OR CVA[tiab] OR TIA[tiab] OR "Cerebrovascular Circulation"[MeSH] OR "Cerebral Circulation"[tiab] OR "Cerebrovascular Circulation"[tiab] OR "Cerebral Blood Flow"[tiab] OR CBF[tiab] OR "Cerebrovascular Disease"[tiab] OR "Cerebrovascular Event"[tiab] OR "Cerebrovascular Disorder"[tiab] OR "Cerebral Vascular Accident"[tiab] OR "Cerebral Vascular Disorder"[tiab] OR "Acute Stroke"[tiab] OR "Acute Ischemic Stroke"[tiab] OR "Cerebral Stroke"[tiab] OR "Cerebral Vascular Ischemia"[tiab] OR "Focal Ischemia"[tiab] OR "Global Ischemia"[tiab] OR "Focal Cerebral Ischemia"[tiab] OR "Global Cerebral Ischemia"[tiab] OR "Hemorrhagic Stroke"[tiab] OR "Subarachnoid Hemorrhage"[tiab] OR "Intracerebral Hemorrhage"[tiab]) AND (rat[Title/Abstract] OR mice[Title/Abstract] OR murine[Title/Abstract] OR rodent[Title/Abstract] OR animal model[Title/Abstract] OR "Animals"[MeSH] OR Animal*[tiab] OR Rats[tiab] OR Mouse[tiab] OR Rabbit*[tiab] OR Primate*[tiab] OR Monkey*[tiab] OR Dog[tiab] OR Canine*[tiab] OR Cat[tiab] OR Feline*[tiab] OR Pig[tiab] OR Swine[tiab] OR Porcine[tiab] OR Sheep[tiab] OR Ovine[tiab] OR Goat*[tiab] OR Caprine[tiab] OR Zebrafish[tiab] OR "In Vitro"[tiab] OR "Ex Vivo"[tiab] OR "Cell Culture"[tiab] OR "Organ Culture"[tiab] OR "In Vitro Techniques"[MeSH] OR "Cells, Cultured"[MeSH] OR "Organ Culture Techniques"[MeSH] OR "Disease Models, Animal"[MeSH] OR "Animal Experimentation"[MeSH] OR "Preclinical Study"[tiab] OR "Experimental Study"[tiab]) AND (infarct*[Title/Abstract] OR deficit*[Title/Abstract] OR impair*[Title/Abstract] OR damage[Title/Abstract] OR protect*[Title/Abstract] OR injur*[Title/Abstract] OR pathophysiology[Title/Abstract] OR pathogenesis[Title/Abstract] OR pathologic[Title/Abstract] OR edema[Title/Abstract] OR haemorrhag*[Title/Abstract] OR hemorrhag*[Title/Abstract] OR lesion*[Title/Abstract] OR ischemi*[Title/Abstract] OR ischaemi*[Title/Abstract] OR neurolog*[Title/Abstract] OR outcome*[Title/Abstract] OR size[Title/Abstract] OR volume[Title/Abstract] OR apoptosis[Title/Abstract] OR autophagy[Title/Abstract] OR inflammation[Title/Abstract] OR oxidative[Title/Abstract] OR necrosis[Title/Abstract] OR signaling[Title/Abstract] OR mitochondrial[Title/Abstract] OR glutamate[Title/Abstract] OR blood brain barrier[Title/Abstract] OR hemorrhage[Title/Abstract] OR vasogenic[Title/Abstract] OR cytotoxic[Title/Abstract] OR edema[Title/Abstract])

Google Scholar:

"berberine" OR isoquinoline OR berberis OR coptis OR bisbenzylisoquinoline AND "ischemia" OR "ischaemia" OR stroke OR infarction OR thrombo* OR emboli* OR ischemic OR ischaemic OR cerebrovasc* OR "cerebral vascular" OR MCAO OR "middle cerebral artery occlusion" OR BCCAo OR "cerebral hypoperfusion" AND "rat" OR mice OR murine OR rodent OR "animal model" AND infarct* OR deficit* OR impair* OR damage OR protect* OR injur* OR pathophysiology OR pathogenesis OR pathologic OR edema OR haemorrhag* OR hemorrhag* OR lesion* OR ischemi* OR ischaemi* OR neurolog* OR outcome* OR size OR volume OR apoptosis OR autophagy OR inflammation OR oxidative OR necrosis OR signaling OR mitochondrial OR glutamate OR "blood brain barrier" OR hemorrhage OR vasogenic OR cytotoxic OR edema

**ScienceDirect:**

(TITLE-ABSTR-KEY(berberine) OR TITLE-ABSTR-KEY(isoquinoline) OR TITLE-ABSTR-KEY(berberis) OR TITLE-ABSTR-KEY(coptis) OR TITLE-ABSTR-KEY(bisbenzylisoquinoline))

AND

(TITLE-ABSTR-KEY(ischemia) OR TITLE-ABSTR-KEY(ischaemia) OR TITLE-ABSTR-KEY(stroke) OR TITLE-ABSTR-KEY(infarction) OR TITLE-ABSTR-KEY(thrombo) OR TITLE-ABSTR-KEY(emboli) OR TITLE-ABSTR-KEY(ischemic) OR TITLE-ABSTR-KEY(ischaemic) OR TITLE-ABSTR-KEY(cerebrovasc) OR TITLE-ABSTR-KEY("cerebral vascular") OR TITLE-ABSTR-KEY(MCAO) OR TITLE-ABSTR-KEY("middle cerebral artery occlusion") OR TITLE-ABSTR-KEY(BCCAo) OR TITLE-ABSTR-KEY("cerebral hypoperfusion"))

AND

(TITLE-ABSTR-KEY(rat) OR TITLE-ABSTR-KEY(mice) OR TITLE-ABSTR-KEY(murine) OR TITLE-ABSTR-KEY(rodent) OR TITLE-ABSTR-KEY("animal model"))

AND

(TITLE-ABSTR-KEY(infarct) OR TITLE-ABSTR-KEY(deficit) OR TITLE-ABSTR-KEY(impair) OR TITLE-ABSTR-KEY(damage) OR TITLE-ABSTR-KEY(protect) OR TITLE-ABSTR-KEY(injur) OR TITLE-ABSTR-KEY(pathophysiology) OR TITLE-ABSTR-KEY(pathogenesis) OR TITLE-ABSTR-KEY(pathologic) OR TITLE-ABSTR-KEY(edema) OR TITLE-ABSTR-KEY(haemorrhag*) OR TITLE-ABSTR-KEY(hemorrhag) OR TITLE-ABSTR-KEY(lesion) OR TITLE-ABSTR-KEY(ischemi) OR TITLE-ABSTR-KEY(ischaemi) OR TITLE-ABSTR-KEY(neurolog) OR TITLE-ABSTR-KEY(outcome) OR TITLE-ABSTR-KEY(size) OR TITLE-ABSTR-KEY(volume) OR TITLE-ABSTR-KEY(apoptosis) OR TITLE-ABSTR-KEY(autophagy) OR TITLE-ABSTR-KEY(inflammation) OR TITLE-ABSTR-KEY(oxidative) OR TITLE-ABSTR-KEY(necrosis) OR TITLE-ABSTR-KEY(signaling) OR TITLE-ABSTR-KEY(mitochondrial) OR TITLE-ABSTR-KEY(glutamate) OR TITLE-ABSTR-KEY("blood brain barrier") OR TITLE-ABSTR-KEY(hemorrhage) OR TITLE-ABSTR-KEY(vasogenic) OR TITLE-ABSTR-KEY(cytotoxic) OR TITLE-ABSTR-KEY(edema))

Europe PMC:

Filter: Research Article

#1 berberine AND ischemia AND (rat OR mice OR rodent OR animal model) AND (infarct* OR deficit* OR impair* OR damage OR injury OR edema OR haemorrhage OR hemorrhage OR lesion* OR ischemia OR ischaemia OR neurological OR outcome OR size OR volume)

#2 berberine AND ischaemia AND (rat OR mice OR rodent OR animal model) AND (infarct* OR deficit* OR impair* OR damage OR injury OR edema OR haemorrhage OR hemorrhage OR lesion* OR ischemia OR ischaemia OR neurological OR outcome OR size OR volume)

#3 isoquinoline AND ischemia AND (rat OR mice OR rodent OR animal model) AND (infarct* OR deficit* OR impair* OR damage OR injury OR edema OR haemorrhage OR hemorrhage OR lesion* OR ischemia OR ischaemia OR neurological OR outcome OR size OR volume)

#4 isoquinoline AND ischaemia AND (rat OR mice OR rodent OR animal model) AND (infarct* OR deficit* OR impair* OR damage OR injury OR edema OR haemorrhage OR hemorrhage OR lesion* OR ischemia OR ischaemia OR neurological OR outcome OR size OR volume)

#5 berberis AND ischemia AND (rat OR mice OR rodent OR animal model) AND (infarct* OR deficit* OR impair* OR damage OR injury OR edema OR haemorrhage OR hemorrhage OR lesion* OR ischemia OR ischaemia OR neurological OR outcome OR size OR volume)

#6 berberis AND ischaemia AND (rat OR mice OR rodent OR animal model) AND (infarct* OR deficit* OR impair* OR damage OR injury OR edema OR haemorrhage OR hemorrhage OR lesion* OR ischemia OR ischaemia OR neurological OR outcome OR size OR volume)

#7 coptis AND ischemia AND (rat OR mice OR rodent OR animal model) AND (infarct* OR deficit* OR impair* OR damage OR injury OR edema OR haemorrhage OR hemorrhage OR lesion* OR ischemia OR ischaemia OR neurological OR outcome OR size OR volume)

#8 coptis AND ischaemia AND (rat OR mice OR rodent OR animal model) AND (infarct* OR deficit* OR impair* OR damage OR injury OR edema OR haemorrhage OR hemorrhage OR lesion* OR ischemia OR ischaemia OR neurological OR outcome OR size OR volume)

#9 bisbenzylisoquinoline AND ischemia AND (rat OR mice OR rodent OR animal model) AND (infarct* OR deficit* OR impair* OR damage OR injury OR edema OR haemorrhage OR hemorrhage OR lesion* OR ischemia OR ischaemia OR neurological OR outcome OR size OR volume)

#10 bisbenzylisoquinoline AND ischaemia AND (rat OR mice OR rodent OR animal model) AND (infarct* OR deficit* OR impair* OR damage OR injury OR edema OR haemorrhage OR hemorrhage OR lesion* OR ischemia OR ischaemia OR neurological OR outcome OR size OR volume)

Open Access Journals (DOAJ):

berberine AND ischemia AND (rat OR mice) AND (infarct* OR deficit* OR impair* OR damage OR injury OR edema OR haemorrhage OR hemorrhage OR lesion* OR ischemia OR ischaemia OR neurological OR outcome OR size OR volume)

berberine AND ischaemia AND (rat OR mice) AND (infarct* OR deficit* OR impair* OR damage OR injury OR edema OR haemorrhage OR hemorrhage OR lesion* OR ischemia OR ischaemia OR neurological OR outcome OR size OR volume)

isoquinoline AND ischemia AND (rat OR mice) AND (infarct* OR deficit* OR impair* OR damage OR injury OR edema OR haemorrhage OR hemorrhage OR lesion* OR ischemia OR ischaemia OR neurological OR outcome OR size OR volume)

isoquinoline AND ischaemia AND (rat OR mice) AND (infarct* OR deficit* OR impair* OR damage OR injury OR edema OR haemorrhage OR hemorrhage OR lesion* OR ischemia OR ischaemia OR neurological OR outcome OR size OR volume)

berberis AND ischemia AND (rat OR mice) AND (infarct* OR deficit* OR impair* OR damage OR injury OR edema OR haemorrhage OR hemorrhage OR lesion* OR ischemia OR ischaemia OR neurological OR outcome OR size OR volume)

berberis AND ischaemia AND (rat OR mice) AND (infarct* OR deficit* OR impair* OR damage OR injury OR edema OR haemorrhage OR hemorrhage OR lesion* OR ischemia OR ischaemia OR neurological OR outcome OR size OR volume)

coptis AND ischemia AND (rat OR mice) AND (infarct* OR deficit* OR impair* OR damage OR injury OR edema OR haemorrhage OR hemorrhage OR lesion* OR ischemia OR ischaemia OR neurological OR outcome OR size OR volume)

coptis AND ischaemia AND (rat OR mice) AND (infarct* OR deficit* OR impair* OR damage OR injury OR edema OR haemorrhage OR hemorrhage OR lesion* OR ischemia OR ischaemia OR neurological OR outcome OR size OR volume)

bisbenzylisoquinoline AND ischemia AND (rat OR mice) AND (infarct* OR deficit* OR impair* OR damage OR injury OR edema OR haemorrhage OR hemorrhage OR lesion* OR ischemia OR ischaemia OR neurological OR outcome OR size OR volume)

bisbenzylisoquinoline AND ischaemia AND (rat OR mice) AND (infarct* OR deficit* OR impair* OR damage OR injury OR edema OR haemorrhage OR hemorrhage OR lesion* OR ischemia OR ischaemia OR neurological OR outcome OR size OR volume)

**Scopus:**

(TITLE-ABS-KEY(berberin*) OR TITLE-ABS-KEY(isoquinoline) OR TITLE-ABS-KEY(coptis) OR TITLE-ABS-KEY(huanglian))

AND

(TITLE-ABS-KEY(stroke) OR TITLE-ABS-KEY("cerebral ischemia") OR TITLE-ABS-KEY("brain ischemia") OR TITLE-ABS-KEY("cerebral infarct*") OR TITLE-ABS-KEY(mcao) OR TITLE-ABS-KEY("middle cerebral artery occlusion") OR TITLE-ABS-KEY(bccao) OR TITLE-ABS-KEY("bilateral carotid artery occlusion") OR TITLE-ABS-KEY(hypoxi* OR ischem* OR infarct* OR thrombo* OR embol*) OR TITLE-ABS-KEY("transient ischemic attack"))

AND

(TITLE-ABS-KEY(neuroprotection) OR TITLE-ABS-KEY("anti-apoptotic") OR TITLE-ABS-KEY(anti-inflamm*) OR TITLE-ABS-KEY(anti-oxidant) OR TITLE-ABS-KEY("signaling pathway*") OR TITLE-ABS-KEY("infarct volume") OR TITLE-ABS-KEY(edema) OR TITLE-ABS-KEY(inflamm*) OR TITLE-ABS-KEY("oxidative stress") OR TITLE-ABS-KEY(mitochondria) OR TITLE-ABS-KEY("blood flow") OR TITLE-ABS-KEY(bbb) OR TITLE-ABS-KEY("blood brain barrier"))

AND

(LIMIT-TO(SRCTYPE,"j") OR LIMIT-TO(SRCTYPE,"p"))

Web of Science

TS=((berberine OR isoquinoline OR "isoquinoline alkaloid" OR berberis OR coptis OR huanglian OR bisbenzylisoquinoline OR jatrorrhizine OR palmatine OR epiberberine OR coptisine OR groenlandicine OR "852-A" OR "berberine hydrochloride" OR "berberine sulfate") AND ("cerebral ischemia" OR "brain ischemia" OR "cerebral infarction" OR "middle cerebral artery occlusion" OR MCAO OR "bilateral carotid artery occlusion" OR BCCAO OR stroke OR isch?emi* OR infarct* OR thrombo* OR embol* OR hypoxi*) AND (neuroprotection OR neuroprotective OR "nerve cell protection" OR anti-apoptotic OR anti-inflamm* OR anti-oxidant OR "signaling pathway*" OR "infarct volume" OR edema OR inflamm* OR "oxidative stress" OR mitochondria OR "blood flow" OR "blood brain barrier") AND (animal* OR rat OR rats OR mouse OR mice OR murine OR rodent* OR rabbit* OR pig OR pigs OR porcine OR primate* OR macaque))

Embase

('berberine'/exp OR berberine OR 'isoquinoline alkaloid'/exp OR 'berberis'/exp OR berberis OR coptis OR huanglian) AND ('cerebral ischemia'/exp OR 'brain ischemia' OR 'cerebral infarction'/exp OR 'middle cerebral artery occlusion':ti,ab OR mcao:ti,ab OR 'bilateral carotid artery occlusion':ti,ab OR bccao:ti,ab) AND ('neuroprotection'/exp OR neuroprotection:ti,ab OR 'anti apoptotic agent'/exp OR 'anti inflammatory agent'/exp OR antioxidant/exp OR 'infarct volume':ti,ab OR edema:ti,ab OR inflamm*:ti,ab OR 'oxidative stress':ti,ab OR mitochondria:ti,ab OR 'blood flow':ti,ab OR 'blood brain barrier':ti,ab) AND (animal/exp OR rat/exp OR mouse/exp OR mice:ti,ab OR murine:ti,ab OR rodent/exp OR rabbit/exp OR pig/exp OR porcine:ti,ab) AND [english]/lim AND [2010-2023]/py

Cochrane library

#1 berberine OR isoquinoline OR berberis OR coptis OR huanglian OR bisbenzylisoquinoline OR jatrorrhizine OR palmatine OR epiberberine OR coptisine OR groenlandicine OR "berberine chloride" OR "berberine sulfate" OR “berberrubine” OR “protoberberine” OR “benzylisoquinoline” OR “benzyltetraisoquinoline” OR “bisbenzylquinolizidine”

#2 ischemi* OR isch?emi* OR infarct* OR cerebrovasc* OR cerebrovascular OR stroke OR “cerebral ischemia” OR “brain ischemia” OR “cerebral infarction” OR “brain infarction” OR “middle cerebral artery occlusion” OR mcao OR “intracranial arterial disease*” OR “intracranial embolism” OR “intracranial thrombosis” OR “cerebral hemorrhage” OR “transient ischemic attack”

#3 #1 AND #2
